# Suicidal behaviour in psychodermatology patients: Identifying characteristics and a new model for referral

**DOI:** 10.1002/ski2.207

**Published:** 2023-05-25

**Authors:** Katie Lockwood, Kirsty Smith, Ruth Taylor, Alia Ahmed

**Affiliations:** ^1^ Department of Dermatology Swansea Bay University Health Board Swansea UK; ^2^ King Edward VII Hospital, Windsor London UK; ^3^ Centre for Psychiatry Wolfson Institute Queen Mary University London London UK

## Abstract

It is well known that skin disease is associated with significant psychosocial morbidity, and that patients with skin disease can present with higher rates of suicidality than the general population. Clinicians often report numerous barriers to detecting and managing suicidality in busy outpatient settings. We aimed to establish the degree of suicidality within our psychodermatology patients and establish key characteristics that may serve as additional risk factors for suicidality. We conducted a retrospective review of clinical letters, patient notes, and a clinical database, for all 69 patients that attended our psychodermatology clinic since it was founded. Two practitioners independently recorded patient baseline demographics, presenting dermatological condition, comorbidities, Dermatology Life Quality Index scores and self‐reported suicidal behaviour for each patient. From this we calculated how many patients displayed signs of active suicidality, and identified common themes and characteristics within this patient group. We went onto develop a flow diagram to guide professionals when faced with an actively suicidal patient in clinic.

## Graphical abstract

We conducted a retrospective observational study to investigate levels of active suicidality in psychodermatology patients. From this we identified identified key risk factors for suicidality and proposed a model for assessing and managing a suicidal patient in clinic.

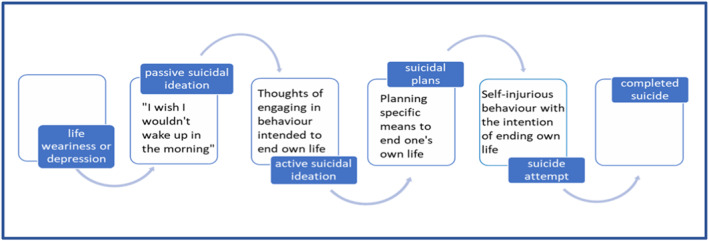

1

**What is already known about this topic?**

- There is a close relationship between skin disease, mental health and quality of life.
- Higher rates of suicidality are observed in patients with skin disease, especially in conditions such as psoriasis, atopic dermatitis and acne.

**What does this study add?**

- Highlights high prevalence of suicidal ideation in psychodermatology patients and identifies some risk factors and patient characteristics associated with suicidality in psychodermatology patients.
- Provides a framework to support clinicians in assessing for suicidality in clinic, and acting swiftly to safely manage and refer onward to psychiatry when this is identified.

## 2 INTRODUCTION

The relationship between skin disease, mental health and quality of life has been well documented^1^ and the recent Getting It Right First Time report highlighted the need to improve access to psychodermatology services^2^ in order to better serve our patients. Dermatology patients can experience a wide range of distressing thoughts and emotions as a consequence of their skin disease and society's reaction to it; one symptom that is essential to recognise and act on is suicidality. Suicidality has been observed at higher rates in dermatology patients^3^; especially in patients with conditions such as psoriasis,^4^ atopic dermatitis^5^ and acne.^6^ For example, Sandhu et al found that patients with atopic dermatitis were 36% more likely to attempt suicide than those without atopic dermatitis.^5^ The World Health Organisation has identified suicide as a leading cause of death worldwide and is working to make suicide prevention a high priority on the global public health agenda.^7^ In line with this, Public Health England have created resources for local suicide prevention planning.^8^ The hospital team is often the main source of support for patients with skin disease, and dermatologists have a key role to play in screening for and detecting signs of low mood and suicidality, in order to help prevent completed suicide.

**FIGURE 1:**
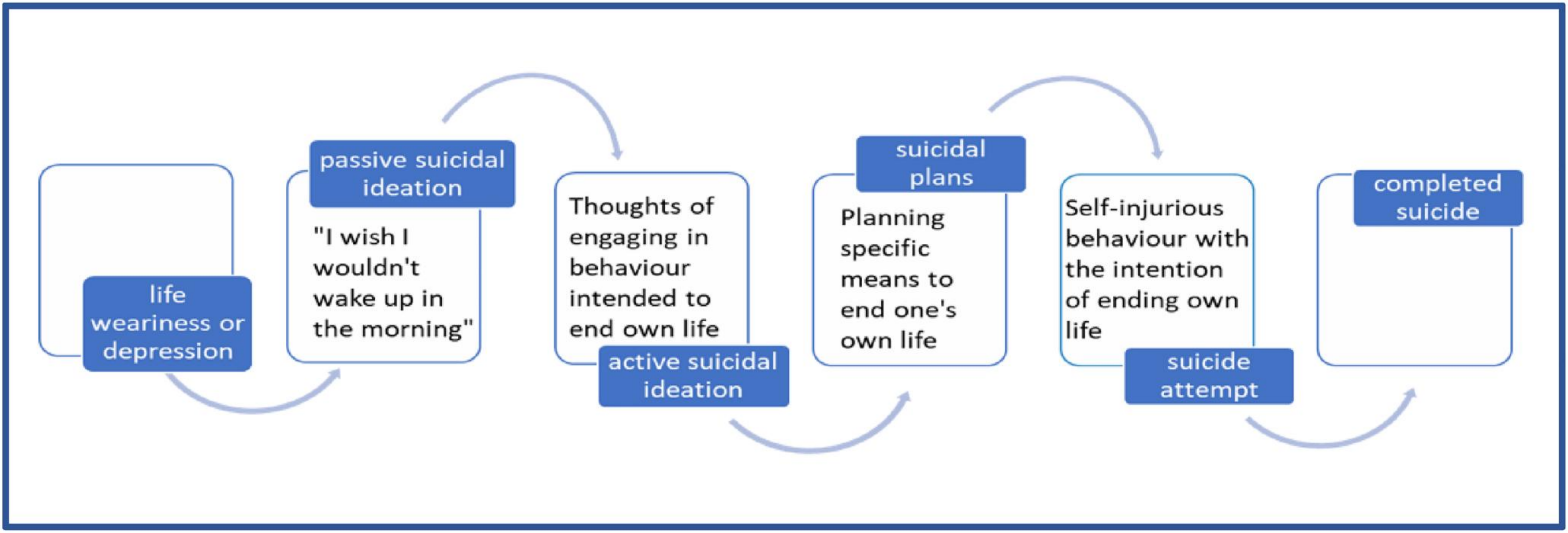
Diagram to demonstrate the continuum of suicidality with increasing risk of leading to completed suicide; although there are exceptions to this rule and some suicidal attempts difficult to predict

When thinking about suicide, ‘suicidal behaviour’ and ‘suicidality’ are broad terms and can be viewed as a spectrum of thoughts and behaviours, as outlined in Figure 1. Acting upon early signs of suicidality may help to prevent the progression to completed suicide. However, a recent study found that healthcare professionals working within specialist psychodermatology settings have limited training on mental health referral pathways and often lack understanding of the signs of suicidal behaviours.^9^ There is also a common misconception that asking about suicidality increases the risk of suicide, when this has not been found to be the case.^10^

Here we present data from our own specialist psychodermatology clinic that pertains directly to suicidal behaviour and highlights relevant patient characteristics that may be related to higher risk of suicide. We describe known risk factors and stress the importance of not relying on screening tools for suicidality. We outline a framework to equip health professionals with a practical action plan, to ensure safety and appropriate referral of dermatology patients where high‐risk suicidal behaviour is identified or suspected.

## 3 PARTICIPANTS AND MATERIALS

We aimed to establish rates of suicidality within our psychodermatology patients, and identify key themes and characteristics that may serve as risk factors. To do so we completed a retrospective analysis of all 69 patients who attended our local adult psychodermatology clinic since its creation. The clinic is run by one consultant dermatologist with a special interest in psychodermatology and background training in psychology. It is supported by the local talking therapies team. The criteria for acceptance is broad; any patient who has a psychodermatology issue as assessed by the referring clinician. The clinic accepts patients from any region across the United Kingdom. All patients complete a Dermatology Life Quality Index (DLQI) and Hospital Anxiety and depression Scale assessment in the waiting room prior to every clinic visit. They are routinely asked about suicidal behaviours as part of their consultation. The consultation proforma includes: presenting complaint, history of presenting complaint, past medical history, medications and allergies, family history (including psychiatric conditions), social history (occupation, home circumstances, alcohol use, substance use, social support network, relationships with family and others), traumatic life‐events, lifestyle factors (diet, sleep, exercise), mood, anxiety, suicidal behaviours and a review of systems. The responses are self‐reported by patients. In situations of clinical relevance a urine toxicology is arranged with patient consent to verify substance use.

Two practitioners independently reviewed each patient's clinical letters, patient notes, and a clinical database. They recorded baseline patient demographics, presenting dermatological condition, comorbidities, DLQI scores and self‐reported suicidal behaviours. From this we calculated the number of patients with evidence of active suicidality and identified common themes and characteristics within this patient group.

## 4 RESULTS

Out of the 69 patients screened we identified 16 with active suicidal ideation (23%). The demographics of the patients with evidence of suicidality are outlined in Table 1. We observed two peak incidences where the risk appeared highest, 25–35 years and 50–69 years. Many patients had recently moved to the UK and thereby had less social support. 69% were without a long term partner, divorced or widowed; again suggesting that social isolation and reduced community support are risk factors for suicidality.

**TABLE 1:** Demographic data for the 16 patients with active suicidal ideation

Patient Demographics	
Gender	63% female, 37% male.
Ethnicity	63% White British.
Age	Incidence peaked in two age groups: 25–35 years (44%) (youngest patients seen). 50–69 years (38%).
Marital status	69% were without a long‐term partner, divorced or widowed.
Employment status	56% were unemployed or on long term sick leave.

Of these 16 there were approximately equal proportions of patients with a cutaneous presentation of primary psychiatric conditions (e.g. dermatitis artefacta, delusional infestation) and patients with suicidal ideation as a complication of dermatological disease (e.g. acne, eczema), 44% and 56% respectively. There was a high degree of reported psychiatric co‐morbidity as outlined in Table 2, along with substance abuse and reports of previous trauma. Psychiatric co‐morbidity remained at 75% even when anxiety and depression, conditions that are commonly found in this patient group, were excluded.

**TABLE 2:** Co‐morbidities observed in patients with suicidal ideation

% of Patients	Associated condition	
100%	Psychiatric comorbidity present	Depression (15)
		Anxiety (14)
		Post‐traumatic stress disorder (4)
		Emotionally unstable personality disorder (4)
		Attention deficit hyperactivity disorder (2)
		Obsessive compulsive disorder (2)
		Autism (1)
		Schizophrenia (1)
81%	Previous trauma (e.g. childhood abuse, police violence, bereavement inc. stillbirth)	
25%	Substance abuse	

*Note*: Psychiatric co‐morbidity remained at 75% even when depression and anxiety, conditions commonly seen in dermatology patients, were excluded.

## 5 DISCUSSION

We aimed to establish the degree of active suicidality within our psychodermatology patients and identify key characteristics that may serve as risk factors. We found a high level of active suicidality and identified some common risk factors, many of which are consistent with those already known to contribute to suicidality.^11^ These included: psychiatric co‐morbidity, social isolation, substance use disorder, as well as trauma. In this patient group active suicidality was identified in a higher proportion of females than males. This is in keeping with previous research suggesting that acts of suicidality such as deliberate self‐harm are more common in females, however completed suicide is more common in males.^12^ It is also recognised that males are less likely to seek help and volunteer suicidal thoughts and feelings^12^ which may account for the lower rates of expressed suicidality seen here.

Suicidality was identified in patients with a range of primary skin diseases as well as those with cutaneous manifestations of primary psychiatric disease. There appeared to be two peak incidences, namely patients aged 25–35 years and those aged 50–69 years. In England and Wales males aged 45–49 years consistently have the highest age‐specific completed suicide rate,^13^ whereas in Scotland this is slightly lower with men aged 35–39 having the highest rate.^14^ London has had the lowest completed suicide rate across the UK for the last 5 years.^13^ Further studies looking at suicidality in dermatology patients across the country are needed to see if the demographics in our patients match national trends or are distinct to this patient group.

Although having an awareness of risk factors that contribute to suicidality is important when assessing suicide risk, the patient's experience of illness is subjective, and we should complete a basic assessment of mood, suicidal thoughts, and behaviours in all patients. Outside of psychiatry, healthcare professionals often receive limited training on how to go about this and understandably lack confidence.^15^, ^16^

**FIGURE 2:**
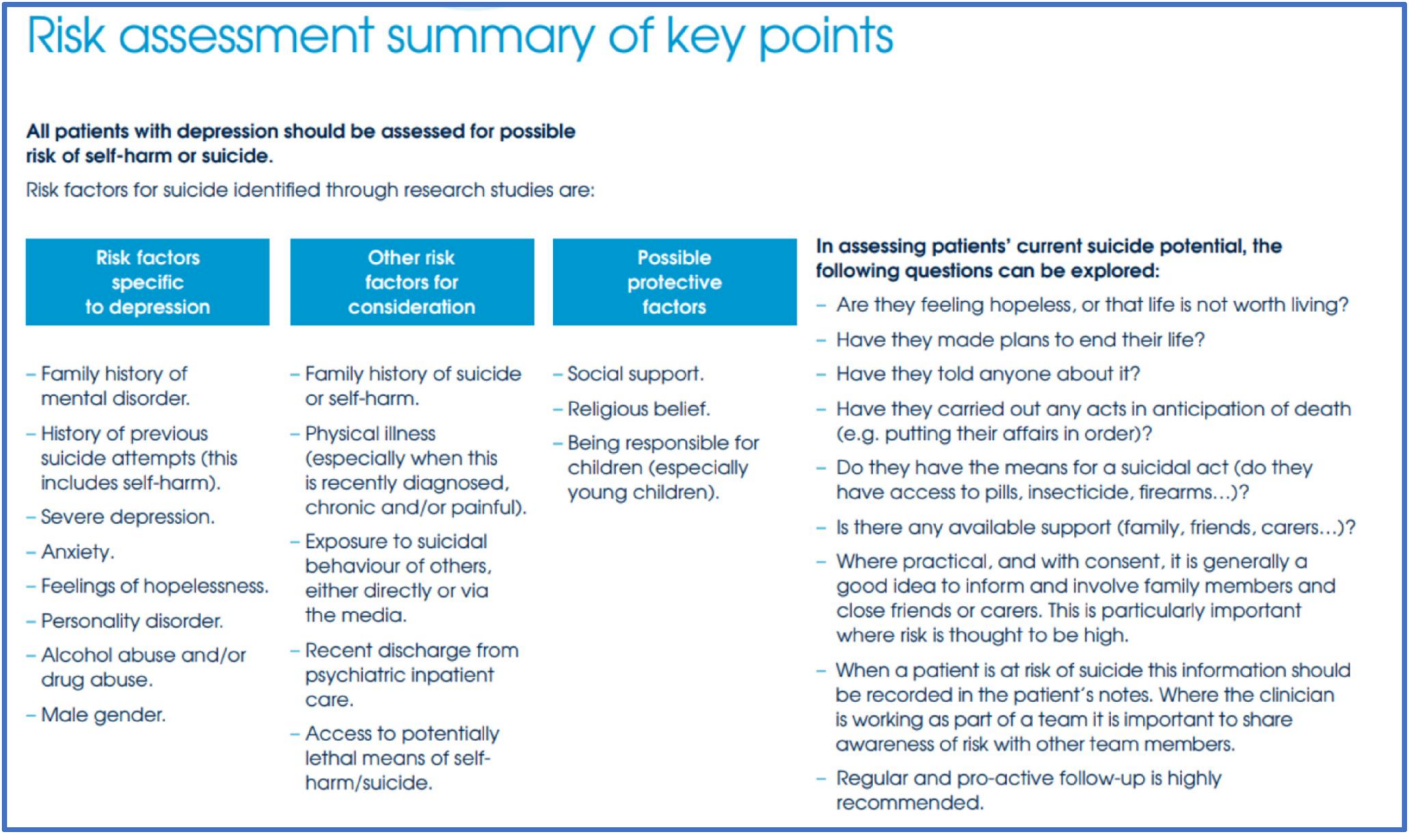
Image used with permission from Centre for suicide research, Department of Psychiatry, University of Oxford. Page 14: *Assessment of suicide risk in people with depression* ^19^

Some helpful questions to introduce the topic of suicidal thoughts are “*Have things ever been so bad, that you have thought that your life is not worth living?*” and “*Have you ever wished you could just not wake up in the morning?*”. If the patient answers ‘Yes’ then questions from here may go onto explore if the patient has ever thought of acting on these feelings (active suicidality), if they have planned how they would end their life (suicidal plan) and what would stop them (protective factors). It is important to give the patient time, validate their feelings and adopt a non‐judgemental approach.^17^ There are no simple effective screening tools to assess suicide risk^18^, ^19^ and staff should train in suicide risk assessment. Even where there are few risk factors, clinicians should not ignore an instinctual feeling that a patient is high risk. Clinicians should also remember that patients may not wish to share all their suicidal feelings and plans. Suicidal risk assessment is not a tick box process and each patient is unique. The University of Oxford Centre for Suicide Research have produced a very helpful simple clinical guide which provides useful information for further assessment of suicide risk^20^ as shown in Figure 2.

**FIGURE 3:**
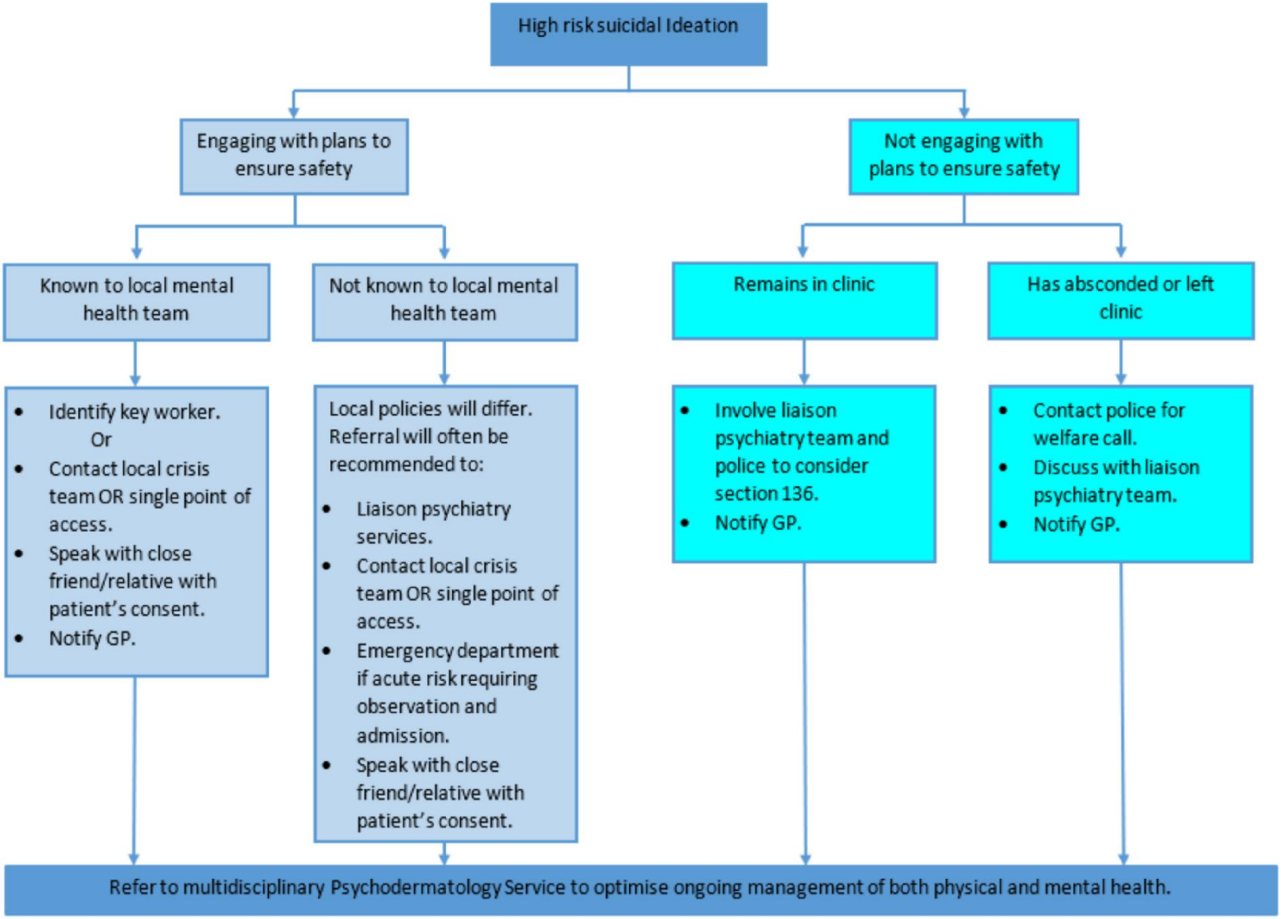
Proposed model for safe referral of patients presenting with high‐risk suicidal ideation

Once suicide risk is identified, it is important to know how to proceed with further management, and whilst we recognise that services may differ depending on location, the flow chart outlined in Figure 3 should provide a helpful guiding framework. Within this framework a high risk patient is any patient who is causing you immediate concern for their safety. They may express suicidal feelings openly or you may have concerns based on information obtained from mental state examination (e.g. signs of self‐neglect, negative thoughts about self) combined with high risk features (outlined in Figure 2).

When faced with this situation in clinic an important starting point is to try and establish if the patient is open to engaging with the healthcare team to ensure their safety. If they are engaging and already known to mental health services, then contacting their key worker or community psychiatric nurse can be helpful. The patient will usually know who this is and the psychiatric liaison team can also help with identifying the appropriate service in the community. If they are not known to services, we would recommend consulting your local trust policy and seeking advice from the psychiatric liaison team. Options from here may include escorting the patient to the emergency department for further assessment or consulting the local crisis team. In both scenarios, if patient consent is given, contacting a close friend or relative to provide additional support is often helpful. It is usually the case that patients are relieved to share that they are experiencing suicidal feelings, are willing to engage in plans to help them, and welcome efforts to make them feel safer.

In the rare situation where the patient is not willing to engage with plans to ensure their safety and you are concerned they pose a risk to themselves or others, you can contact the psychiatric liaison team, and in some cases the police, who have the power to detain a patient under section 136 of the mental health act and take them to a place of safety for further assessment. Should the patient leave the department, again contacting the police to request a welfare check and discussing with the local liaison psychiatry team are helpful options. Almost all general hospitals have psychiatric liaison teams who can be contacted via switchboard and are usually based in A&E. In the rare instances in which there are no on‐site psychiatric liaison services, psychiatry advice will be available by contacting the psychiatrist on call though switchboard. Dermatologists working in psychodermatology clinics should seek out their local psychiatric liaison team and familiarise themselves with the service offered. Psychiatric liaison teams will be very willing to provide advice and information on the best way to seek help for a patient with suicidal ideas.

In all scenarios it is good practice to update the patient's General Practitioner and where possible refer to a psychodermatology service to optimise ongoing management of both physical and mental health.

We acknowledge the limitations of this study including the relatively small sample size from a single Centre, along with the retrospective observational design and the self‐report nature of the data reviewed. We have highlighted some factors that may increase risk of suicidality in our patients but are unable to draw conclusions regarding causality or establish exactly what leads some patients with skin disease to express suicidal thoughts and behaviours. Previous research has found high levels of early maladaptive schemas, ingrained cognitive and emotional patterns, in patients with chronic skin diseases such as eczema and psoriasis. Schemas influence the way a person thinks about themselves, interprets new information and interacts with the world. Schemas relating to vulnerability to harm, defectiveness/shame and social isolation were found to be especially high^21^ and may help to explain the levels of suicidality found here. For example, patients with the defectiveness/shame schema are often convinced that they are worthless and unloveable, feeling shame around perceived flaws and avoiding close social interactions.^22^ They are more likely to pay attention to information that fits in with this schema and interpret new information in line with it. Stigma and stress caused by flares of skin disease contribute to high psychological burden, which combined with schema‐driven maladaptive coping results in further distress.^21^

This study looked purely at patients in the psychodermatology clinic rather than dermatology outpatients as a whole. Although one may expect a higher degree of psychiatric comorbidity and suicidality in patients seen within the psychodermatology clinic, it is important to recognise that these patients can present within any dermatology clinic and indeed are often seen in a general clinic before onward psychodermatology referral is made.

In conclusion, almost a quarter of patients in our psychodermatology clinic were found to have active suicidal ideation. Risk assessment is challenging and many health professionals feel ill equipped to identify and manage the complex mental health needs of our patients. We have stressed the importance of training in risk assessment and not ignoring an instinctual feeling that the patient is high risk. We have outlined a model for safe referral of patients presenting with active suicidality in dermatology outpatient clinics. Further studies looking at suicidality in dermatology outpatients from a range of centres across the UK are needed to further evaluate rates of suicidality and identify common risk factors.

## CONFLICT OF INTEREST

None to declare.

## AUTHOR CONTRIBUTIONS

**Katie Lockwood**: Writing – original draft (Lead); Writing – review & editing (Lead). **Kirsty Smith**: Formal analysis (Equal); Investigation (Equal). **Ruth Taylor**: Conceptualization (Equal); Writing – original draft (Supporting); Writing – review & editing (Supporting). **Alia Ahmed**: Conceptualization (Lead); Formal analysis (Equal); Investigation (Equal); Supervision (Lead); Writing – original draft (Supporting); Writing – review & editing (Supporting).

## ETHICS STATEMENT

Not applicable.

## Data Availability

The data that support the findings of this study are available from the corresponding author upon reasonable request.
